# *GmSNAT1* enhances cold tolerance in soybean by promoting melatonin biosynthesis and interacting with GmPSYR1

**DOI:** 10.1093/plphys/kiag516

**Published:** 2026-07-15

**Authors:** Chunyuan Ren, Tong Cheng, Wenjie Zhang, Suyu Chen, Shaoze Zhang, Liang Cao, Qiang Zhao, Yuxian Zhang, Gaobo Yu

**Affiliations:** College of Agriculture, Heilongjiang Bayi Agricultural University, 163319 Daqing, Heilongjiang, China; College of Agriculture, Heilongjiang Bayi Agricultural University, 163319 Daqing, Heilongjiang, China; College of Life Science and Biotechnology, Heilongjiang Bayi Agricultural University, 163319 Daqing, Heilongjiang, China; College of Agriculture, Heilongjiang Bayi Agricultural University, 163319 Daqing, Heilongjiang, China; College of Agriculture, Heilongjiang Bayi Agricultural University, 163319 Daqing, Heilongjiang, China; College of Agriculture, Heilongjiang Bayi Agricultural University, 163319 Daqing, Heilongjiang, China; College of Agriculture, Heilongjiang Bayi Agricultural University, 163319 Daqing, Heilongjiang, China; College of Agriculture, Heilongjiang Bayi Agricultural University, 163319 Daqing, Heilongjiang, China; National Coarse Cereals Engineering Research Center, Daqing 163319, Heilongjiang, China; College of Horticulture and Landscape Architecture, Heilongjiang Bayi Agricultural University, 163319 Daqing, Heilongjiang, China

## Abstract

The *GmSNAT1* gene, which encodes a key enzyme involved in soybean melatonin biosynthesis, is crucial for abiotic stress tolerance. In the present study, the molecular mechanism by which *GmSNAT1* enhances cold tolerance is elucidated. The cold tolerance of plants was significantly increased by *GmSNAT1* overexpression and reduced by CRISPR/Cas9-mediated knockout, a phenotype that was effectively rescued by exogenous melatonin. Integrated transcriptomic, physiological, and biochemical analyses revealed that the *GmSNAT1*-mediated melatonin pathway activates calcium signaling; coordinates the crosstalk between auxin, abscisic acid, and ethylene; and mobilizes transcription factor networks to orchestrate bidirectional physiological responses. Additionally, the activation of antioxidant systems for reactive oxygen species scavenging and the upregulation of photosynthesis-related genes to maintain photosynthetic stability were explored. The physical interaction between GmSNAT1 and the plant sulfotyrosine peptide receptor GmPSYR1 was confirmed using co-immunoprecipitation, bimolecular fluorescence complementation, and yeast 2-hybrid assays. This interaction may be involved in cold stress signal transduction, regulation of root development, and redox homeostasis through GmPSYR1. Collectively, these findings demonstrate that cold adaptation in soybeans is synergistically enhanced by *GmSNAT1* via a multidimensional axis encompassing melatonin synthesis, signal transduction, and physiological protection, thereby providing a novel molecular target for breeding cold-tolerant crops.

## Introduction

The increasing frequency of global extreme weather events threatens food security and constrains the sustainable development of agriculture (Xiong et al. 2022). Enhancing plant growth under cold conditions remains a critical focus in agricultural research. Sudden, short-term cold stress (0 to 10 °C) substantially affects agricultural production by inhibiting plant growth and reducing yields (El-Mahdy et al. 2024). Each developmental stage of a plant exhibits a distinct optimal temperature range. Growth is arrested when the temperature falls below the growth threshold; severe conditions cause plant damage, ultimately diminishing crop yield (Jan et al. 2024). Cold stress during seed germination can reduce seedling emergence rate and vigor, delay emergence, and disrupt normal crop phenology (Chen and Penfield 2018; Hongchao et al. 2023).

Soybean (*Glycine max*), a vital oilseed and protein-rich feed crop (∼40% protein and ∼20% oil), is cultivated globally (Guo et al. 2022; Duan et al. 2023). Due to intensifying climate change, soybeans are increasingly exposed to cold stress at various growth stages, with the germination and seedling establishment phases being particularly vulnerable (Tsegaw et al. 2023). Cold stress typically induces oxidative stress in plants, which subsequently suppresses photosynthesis (Zhou et al. 2025). The function of organelles, such as chloroplasts and mitochondria, is disrupted, leading to excessive accumulation of reactive oxygen species (ROS) in plant tissues. ROS overload negatively affects cellular, physiological, and biochemical processes, including photosynthesis (Wu et al. 2017; Waszczak et al. 2018). Concurrently, low-temperature conditions substantially alter the chloroplast structure, reduce the activity of key Calvin cycle enzymes, inhibit linear electron transport, and limit the synthesis of photosynthetic products, resulting in ROS production (Crosatti et al. 2013; Song et al. 2021; Soualiou et al. 2022). Upon sensing cold signals, plants rapidly activate signal transduction pathways, triggering a cascade of physiological and biochemical responses that facilitates acclimation. Under cold stress, reduced plasma membrane fluidity activates calcium ion (Ca^2+^) signaling. This signal is transduced via cascades that modulate hormone-mediated metabolism and physiology, regulating plant development (Ding et al. 2019; Ding et al. 2024). Plant antioxidant defense mechanisms are activated to scavenge excess ROS and restore cellular homeostasis (Wang et al. 2024a). Therefore, understanding the physiological mechanisms underlying cold injury and adaptation is conducive to mitigating the adverse effects of cold stress on soybean growth and yield.

Melatonin is an important endogenous plant growth regulator that is crucial for plant stress resistance (Arnao and Hernández-Ruiz 2019). Melatonin enhances plant cold stress tolerance and mitigates the damage resulting from cold stress (Song et al. 2023). Notably, serotonin N-acetyltransferase (SNAT), a rate-limiting enzyme in melatonin biosynthesis, is essential for plant adaptation to cold stress (Byeon et al. 2014; Yang et al. 2021; Kumar et al. 2022). The transcription factor (TF) network governing plant cold-stress responses is also partially regulated by melatonin (Shi et al. 2015; Lee and Back 2016; Wang et al. 2024b). Our previous study revealed that the expression of most soybean B3 TF family members under cold stress was regulated by melatonin (Ren et al. 2023). Furthermore, multiple studies have confirmed that melatonin orchestrates multifaceted transcriptional reprogramming during cold defense, including the upregulation of cold-responsive TFs, for example, C-repeat binding factors (CBFs), activation of antioxidant-related TFs, and suppression of TFs associated with jasmonic acid catabolism (Bajwa et al. 2014; Li et al. 2023).

Plant peptides are novel signaling molecules that trigger intercellular communication to coordinate plant development and stress tolerance (Segonzac and Monaghan 2019; Zhang et al. 2021). These diverse peptides orchestrate growth and stress responses (Xiao et al. 2025). Notably, plant sulfotyrosine peptide (PSY) and its receptor PSYR1 (PSYR) are essential regulators of cell proliferation and stress adaptation (Nguyen and Kim 2023). PSY modulates plasma membrane H^+^-ATPase activity, which provides the energy for active transporters involved in stomatal regulation and cell elongation. This modulation represents a key mechanism by which plants acclimate to environmental stressors (Falhof et al. 2016; Zhang et al. 2024). As the cognate receptor for PSY, PSYR is likely involved in modulating plant responses to abiotic stresses, such as cold and salinity (Nguyen and Kim 2023).

In this study, we revealed a regulatory mechanism for melatonin-enhanced cold tolerance in soybeans by integrating endogenous and exogenous signaling. Exogenous melatonin activates *GmSNAT1* expression, leading to a substantial increase in endogenous melatonin levels, which confers cold tolerance. A signaling cascade is initiated by the *GmSNAT1*-melatonin axis, involving Ca^2+^-mediated transduction, crosstalk among the auxin, abscisic acid (ABA), and ethylene (ETH) pathways, and enhanced endogenous melatonin and auxin biosynthesis. These coordinated responses contribute to improved cold adaptation, particularly by reinforcing root development and vegetative growth under stressful conditions. A novel physical interaction between GmSNAT1 and the sulfotyrosine peptide receptor GmPSYR1 was identified. The activation of *GmPSYR1* promotes the development of cold-tolerant roots and coordinates with the GmSNAT1 pathway to regulate cold adaptation. Furthermore, the interaction between GmSNAT1 and GmPSYR1 may play a critical role in stress signal transduction.

## Results

### Melatonin application enhanced the cold tolerance of different soybean seedling varieties

**Figure 1:**
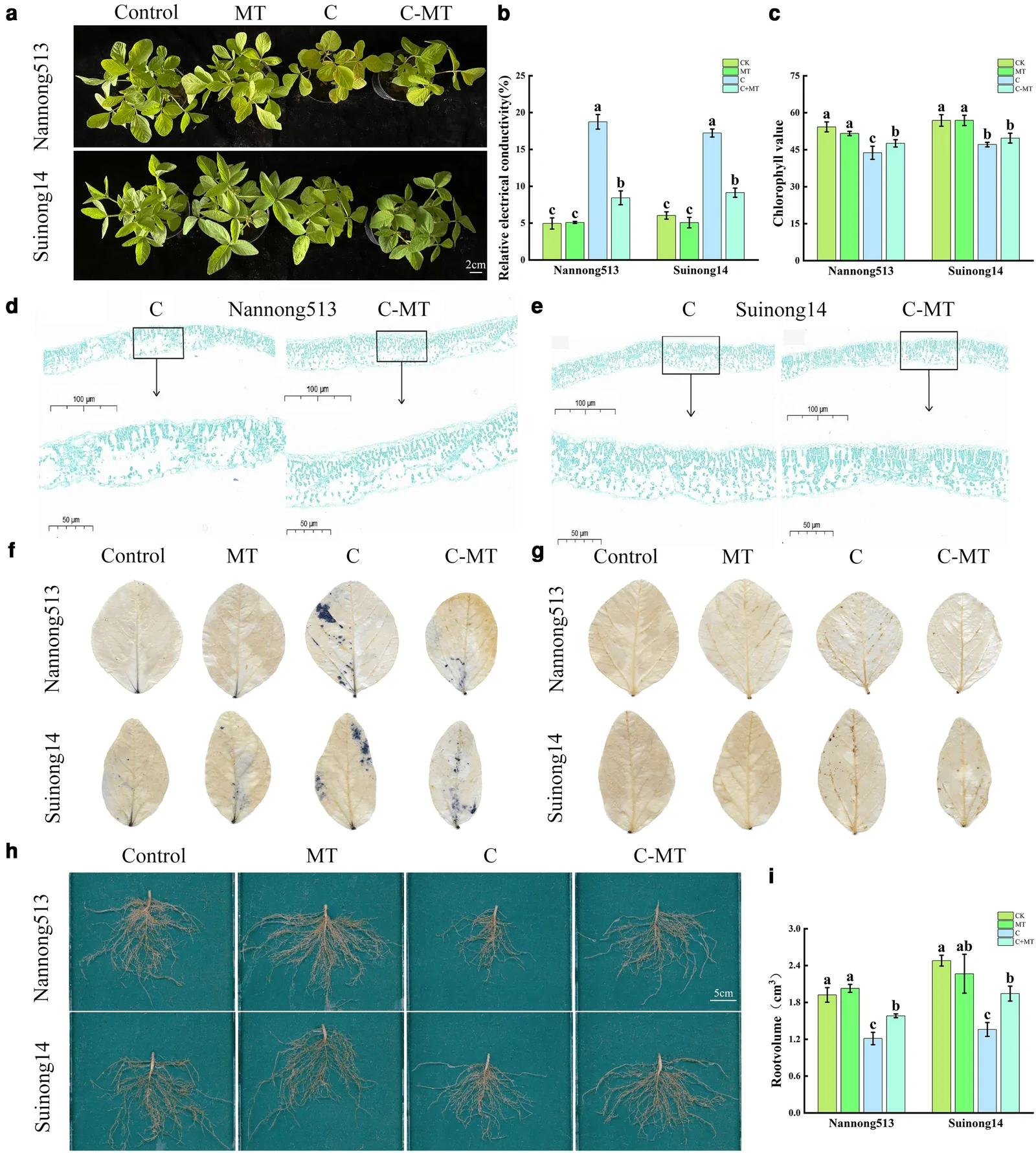
Melatonin-mediated enhancement of cold tolerance in soybean seedlings. (a) Phenotypes of soybean cultivars subjected to cold stress with and without MT. (b) Leaf chlorophyll content (SPAD value) under cold stress. (c and d) Anatomical structure of seedling leaves under cold stress. (e) Superoxide localization (NBT staining). (f) H_2_O_2_ localization (DAB staining). (g) Relative electrolyte leakage (leaf membrane integrity). (h) Representative scan of root architecture. (i) Root volume quantification. CK: control. MT: melatonin treatment. C: 4 °C cold treatment. C-MT: melatonin pretreatment + 4 °C cold treatment. Data are presented as mean ± Sd (*n* = 3 biological replicates). Different letters indicate significant differences (*P* < 0.05, Duncan's test).

The efficacy of melatonin in mitigating cold damage in soybean seedlings has been previously confirmed (Ren et al. 2023). In the present study, the cold-sensitive cultivar ‘Nannong 513’ and cold-tolerant cultivar ‘Suinong 14’ were used to elucidate the role of melatonin in enhancing the growth of soybean seedlings under low-temperature stress (Fig. 1). Soybean leaves were severely damaged by cold stress (Fig. 1b and c), with a substantial accumulation of superoxide (O_2_^−^) and hydrogen peroxide (H_2_O_2_) induction (Fig. 1f and g), alongside a chlorophyll content reduction (Fig. 1c). Palisade mesophyll tissues exhibited a loose and irregular arrangement (Fig. 1d and e; Fig. S1). The subterranean analysis revealed that root growth was markedly inhibited (Fig. 1h and i; Fig. S2). However, melatonin treatment (MT) alleviated cold-induced growth suppression (Fig. 1a and h), reduced ROS accumulation (Fig. 1f and g), and restored the tightly organized palisade tissue structure (Fig. 1d and e). Collectively, these results demonstrate that cold stress in soybean seedlings was mitigated by melatonin, as evidenced by the preservation of photosynthetic integrity and improved growth.

### Exogenous melatonin regulated endogenous melatonin synthesis to enhance antioxidant and photosynthetic systems

Transcriptomic analysis was performed to elucidate the biological mechanisms underlying melatonin-mediated cold tolerance. Gene ontology (GO) enrichment analysis revealed that differentially expressed genes (DEGs) in the melatonin-treated group were significantly enriched in redox regulation, cold response, and ABA signaling pathways compared to those in the cold-only group (Fig. S3). The Kyoto Encyclopedia of Genes and Genomes (KEGG) analysis indicated that the DEGs were enriched in plant hormone signal transduction, mitogen-activated protein kinase (MAPK) signaling, and flavonoid biosynthesis pathways (Fig. S4). Physiological assays demonstrated that under cold stress, Ca^2+^ content in seedlings was increased by 29.1% with the application of melatonin (Fig. S5). This suggests that stress-response activation occurred via Ca^2+^ signaling.

**Figure 2:**
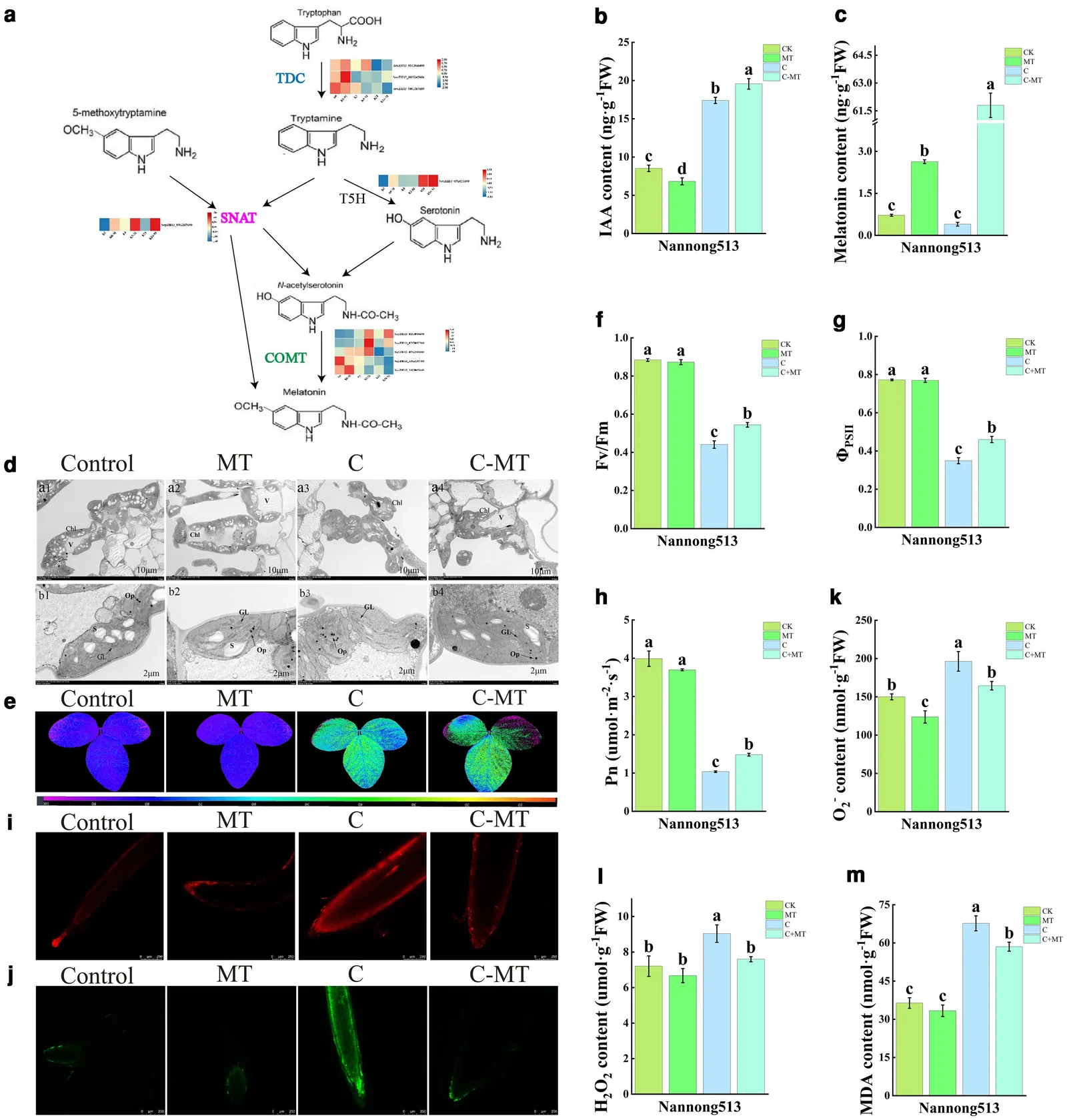
Melatonin-enhanced cold tolerance via calcium ion (Ca^2+^) signaling-mediated regulation of antioxidant and photosynthetic systems. (a) DEG enrichment in the melatonin biosynthesis pathways. (b) Endogenous auxin content. (c) Melatonin content in soybean leaves. (d) Mesophyll cell ultrastructure: a1 to a4, cross-sections (×1,000); b1 to b4, chloroplast details (×5,000). (chl, chloroplast; v, vacuole; gl, grana lamellae; s, starch grain; op, osmophilic plastoglobuli). (e) In vivo chlorophyll fluorescence imaging (representative pseudo-color image). (f to h) Photosynthetic parameters: maximum PSII quantum yield (Fv/Fm), actual PSII efficiency (ΦPSⅡ), and net photosynthetic rate (Pn). (i and j) ROS visualization: superoxide (O_2_^.−^) was detected using DHE staining; H_2_O_2_ was detected using DCFH-DA staining. (k to m) Quantification of O_2_^−^, H_2_O_2_ content, and malonaldehyde content. CK: control. MT: melatonin treatment. C: 4 °C cold treatment. C-MT: melatonin pretreatment + 4 °C cold treatment. Data are presented as mean ± Sd (*n* = 3 biological replicates). Different letters indicate significant differences (*P* < 0.05, Duncan's test).

Under cold stress, key melatonin biosynthesis genes [SNAT and caffeic acid O-methyltransferase {COMT}] were upregulated by exogenous melatonin (Fig. 2a), leading to a 154.65-fold increase in melatonin levels in the soybean leaves (Fig. 2c). However, only a modest increase in auxin levels was observed (Fig. 2b). In the early stages of cold stress (0 to 24 h), transcriptomic analysis of the photosynthetic and antioxidant systems showed melatonin-induced gene upregulation. This includes those involved in carbon fixation (*SoyZH13_02G117000* and *SoyZH13_12G071800*), glucose metabolism (*SoyZH16_02G041200*), chlorophyll synthesis (*SoyZH13_01G223500*), and photosystem I (*SoyZH13_10G022970*) (Fig. S6). Concurrently, key antioxidant genes, for example, the superoxide dismutase (SOD) gene *SoyZH13_10G178300* and the peroxidase (POD) gene *SoyZH13_20G155500*, were upregulated (Fig. S7). In the late stage of cold stress (4 d), the physiological level of soybeans shifted more prominently. The corresponding physiological data confirmed enhanced photosynthetic stability and capacity (Fig. 2d to h), alongside increased antioxidant enzyme activity and nonenzymatic antioxidant levels (Fig. S8). This condition led to the suppression of ROS accumulation, resulting in substantial reductions in O_2_·^−^ and H_2_O_2_ (Fig. 2i to m). Melatonin coordinates signal transduction and energy metabolism to maintain physiological homeostasis in plants under cold-stress conditions.

### *GmSNAT1* expression regulated soybean melatonin biosynthesis

**Figure 3:**
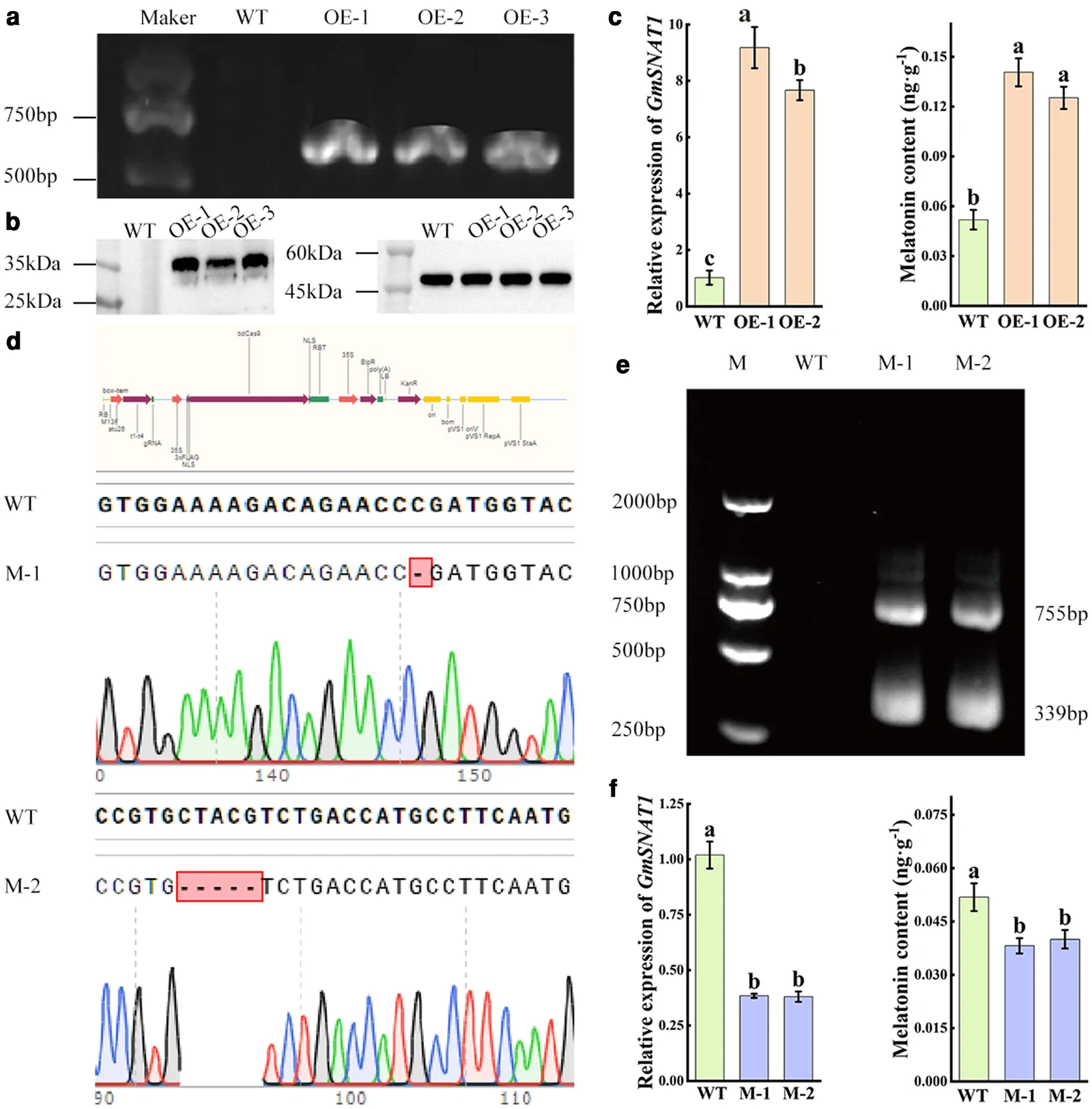
*GmSNAT1*-regulated melatonin biosynthesis in soybeans. (a) PCR genotyping of *GmSNAT1*-OE transgenic lines (688-bp target fragment). (b) Western blot verification: anti-FLAG (for the GmSNAT1 fusion protein) and anti-ACTIN as a loading control were used. (c) *GmSNAT1* transcript levels (qRT-PCR, left axis) and leaf melatonin content (right axis) in WT and OE lines. (d) CRISPR-Cas9 vector design (top) and Sanger sequencing chromatograms of *GmSNAT1* target sites in the WT and knockout mutants (M1 and M2). (e) Screening of positive seedlings of *GmSNAT 1* gene M lines by PCR identification with 2 pairs of Cas9 vector primers (f) *GmSNAT1* transcript abundance (qRT-PCR, left axis) and melatonin content (right axis) in WT and M lines (M1 and M2). Data are presented as mean ± Sd (*n* = 3 biological replicates). Different letters indicate significant differences (*P* < 0.05, Duncan's test).

To functionally characterize *GmSNAT1*, transgenic *GmSNAT1*-overexpressing (OE) lines were generated on a Williams 82 background (Fig. 3a and b). RT-qPCR analysis confirmed that the *GmSNAT1* transcript level in the OE lines was 7.52- to 9.01-fold higher than that in the wild-type (WT) controls (Fig. 3c). This was concomitant with a 2.42- to 2.71-fold increase in leaf melatonin content (Fig. 3e). Using CRISPR-Cas9, 2 independent knockout lines (*GmSNAT1*-M1 and *GmSNAT1*-M2) were obtained (Fig. 3d and e). Reduced *GmSNAT1* expression (37.68% and 37.31% of the WT levels, respectively) was observed in these mutants, accompanied by a corresponding decrease in melatonin content (26.39% and 22.84%, respectively) (Fig. 3f). The validated genetic materials enabled subsequent functional analyses.

### *GmSNAT1* regulated soybean hormone and TF network under cold stress

**Figure 4:**
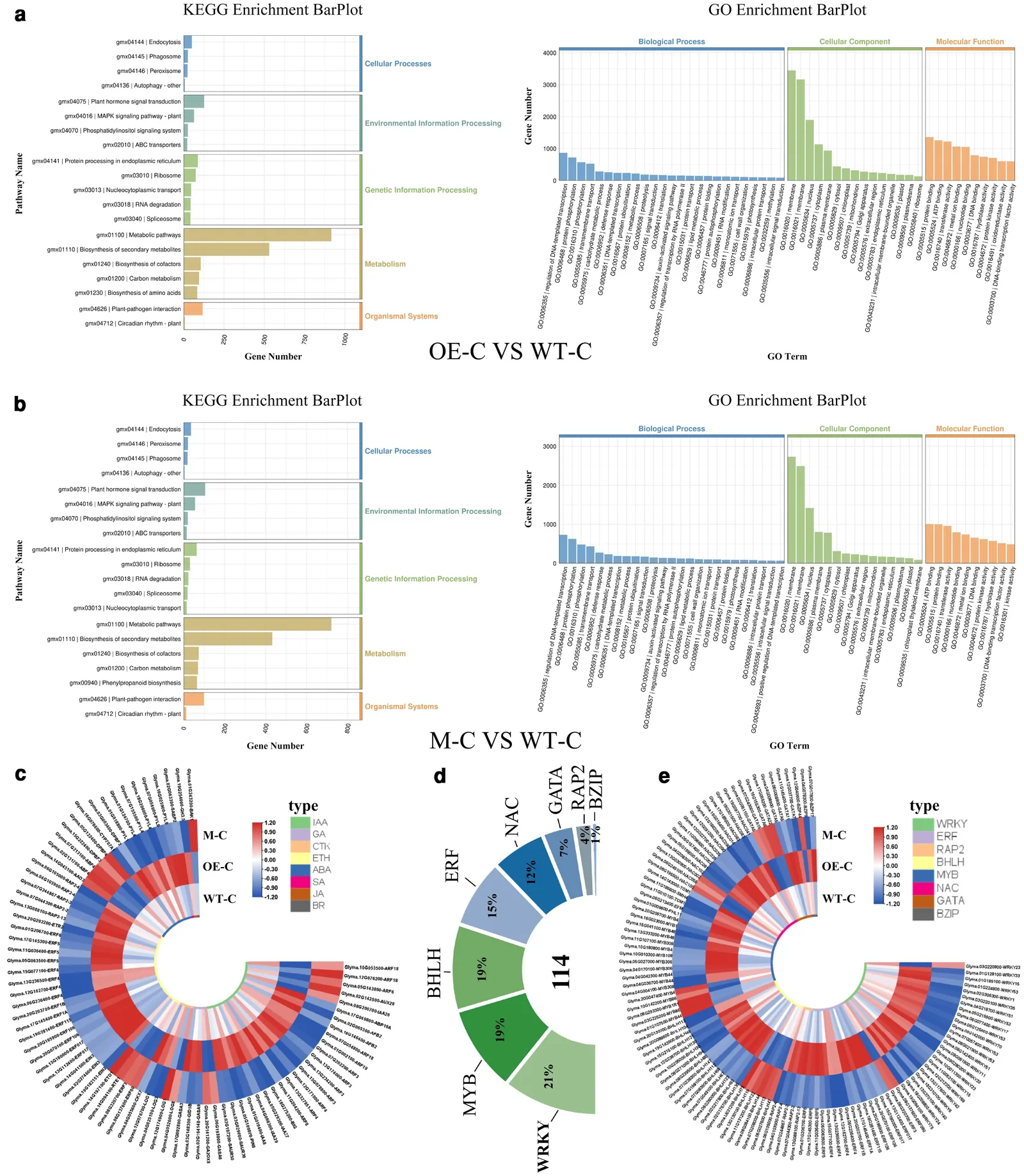
Transcriptomic profiling of transgenic soybean lines under cold-stress conditions. (a) Dual KEGG/GO enrichment of DEGs in *GmSNAT1*-OE vs. WT (OE-C vs. WT-C; C: 4 °C treatment). (b) Dual KEGG/GO enrichment of DEGs in *GmSNAT1*-M vs. WT (M-C vs. WT-C). (c) Heatmap of DEG expression in hormone pathways: signaling pathways of abscisic, jasmonic, and salicylic acids, ETH, auxin, gibberellin, and brassinolide. (d) Quantification of the differentially expressed TFs by family. (e) Heatmap of TF family expression: Expression patterns of WRKY, ERF, BHLH, MYB, NAC, bZIP, GATA, and RAP2 families are shown.

RNA-seq analysis of the transgenic lines cultivated under cold stress was performed to elucidate the role of *GmSNAT1* in cold tolerance. Compared to WT plants, DEGs in knockout (M) and OE lines were significantly enriched in cellular metabolism, plant hormone signaling, MAPK cascades, and signal transduction pathways (Fig. 4a and b). Several genes associated with plant hormone metabolism were enriched and analyzed. Significant differences were observed in the expression patterns of genes related to the auxin, gibberellin, cytokinin, ABA, ETH, salicylic acid, jasmonic acid, and brassinolide signaling pathways between overexpressed and edited lines, with a greater number of DEGs being upregulated in the OE lines (Fig. 4c). The expression of TF families, including WRKY, ERF, bHLH, MYB, NAC, bZIP, GATA, and RAP, was markedly altered at low temperatures (Fig. 4d). The strikingly differential TF expression patterns between the M and OE lines (Fig. 4e) suggest that endogenous melatonin levels critically modulated transcriptional reprogramming during cold stress.

### *GmSNAT1* enhanced soybean cold tolerance by promoting antioxidant defense and photosynthetic homeostasis

Relevant physiological indices were measured, RNA-seq enrichment analysis was performed, and the underlying mechanism was further validated using exogenous melatonin supplementation to elucidate the biological function of *GmSNAT1* in cold tolerance of soybeans.

**Figure 5:**
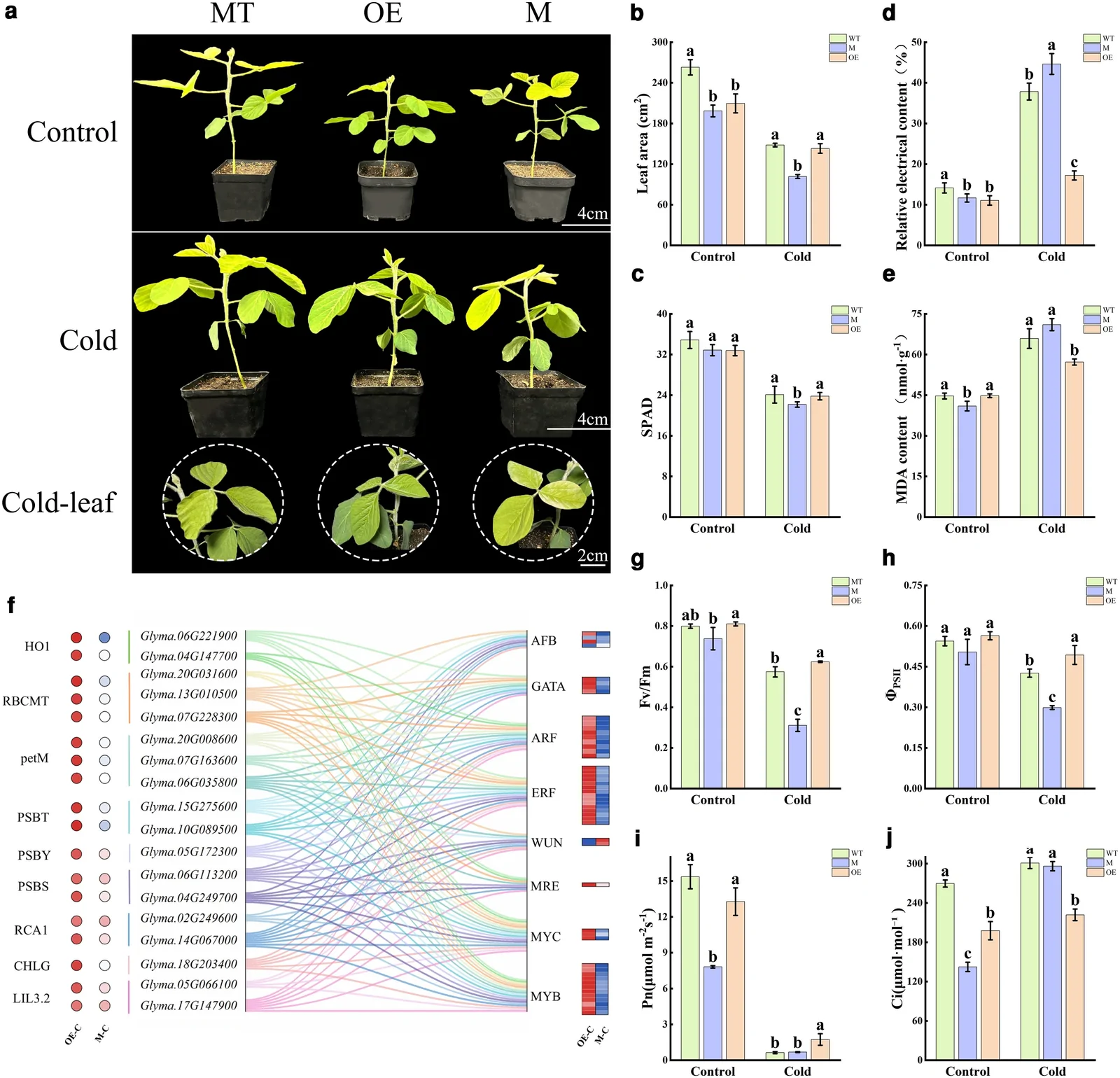
Physiological and molecular responses of *GmSNAT1*-OE and *GmSNAT1*-M soybean plants. (a) Phenotypes of soybean seedlings subjected to low-temperature treatment (4 °C). (b) Leaf area per seedling. (c) SPAD values of leaves. (d) Relative electrolyte conductivity (REC) of leaves. (e) MDA content of leaves. (f) Enrichment analysis of photosynthesis-related DEGs. Left panel: expression levels of key photosynthesis-related genes. Right panel: TFs targeting the promoters of these genes, with their expression levels displayed in the adjacent heatmap. (g) The maximum photochemical efficiency of PSII (Fv/Fm). (h) Actual photochemical efficiency of PSII (Φ_PSII_). (i) Net photosynthetic rate. (j) Intercellular CO_2_ concentration. Data are presented as mean ± Sd (*n* = 3 biological replicates). Different letters indicate significant differences (*P* < 0.05, Duncan's test).

Significantly enhanced low-temperature adaptability was exhibited by OE, with the electrolyte leakage rate (REC) and malondialdehyde (MDA) content being markedly lower than those in the WT plants (Fig. 5a to e). Analysis of root morphology revealed that soybean root growth was inhibited at low temperatures. In contrast, the OE lines maintained strong root growth potential, with significantly greater root length and more root tips than the WT and M lines, despite a slight reduction in average root diameter (Fig. S9). The RNA-seq analysis indicated that most cold-responsive and antioxidant-related genes were upregulated in the OE lines (Fig. S10). Physiological data corroborated this transcriptional pattern. Under low-temperature stress, the activities of SOD, catalase (CAT), and ascorbate peroxidase (APX) were significantly elevated in OE plants (Fig. S11).

In contrast, *GmSNAT1*M plants displayed a cold-sensitive phenotype. Low temperatures significantly reduced the SPAD value and leaf area in the M lines (Fig. 5b and c) and increased the REC and MDA content (Fig. 5d and e). The expression of most of the cold-responsive and antioxidant-related genes was notably downregulated in M plants (Fig. S10), and the activities of POD and APX significantly decreased (Fig. S11).

Enrichment analysis of photosynthesis-related genes demonstrated that the expression levels of these genes were higher in the OE lines than in the M lines (Fig. 5f). Measurements of chlorophyll fluorescence and gas exchange parameters further indicated that the photosynthetic system in OE plants remained highly stable under cold-stress conditions (Fig. 5g to j). Exogenous melatonin supplementation experiments confirmed that *GmSNAT1* regulates low-temperature antioxidant responses and photosynthetic stability of soybeans by modulating endogenous melatonin levels (Figs. S12 to S14). *GmSNAT1* overexpression enhanced antioxidant defense capacity and maintained photosynthetic homeostasis, promoting root growth and improving cold tolerance in soybean seedlings.

### GmSNAT1 interacted with GmPSYR1 to enhance cold signal transduction

**Figure 6:**
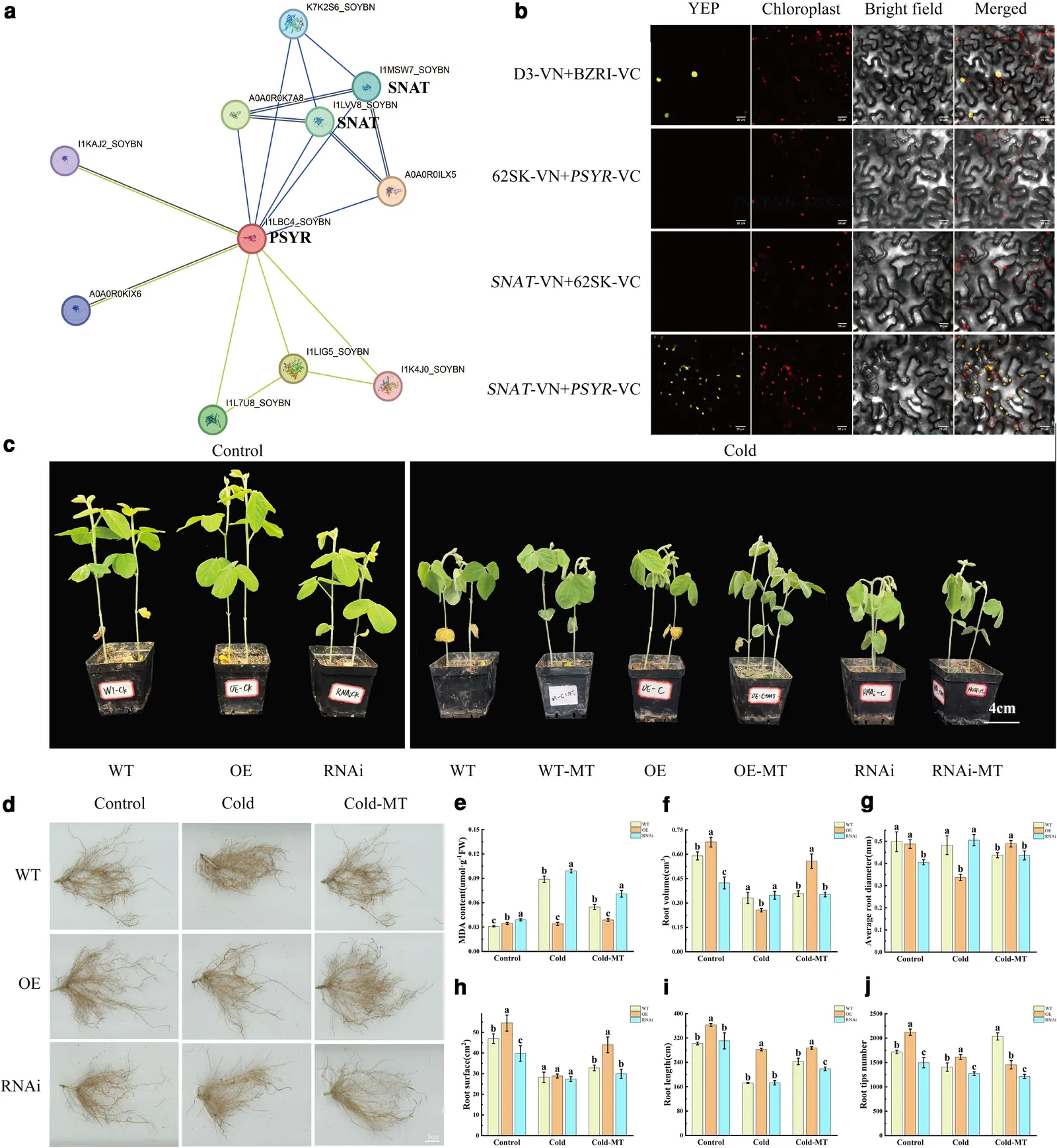
Interaction between GmSNAT1 and PSYR and their regulatory effects on soybean cold tolerance. (a) The protein interaction network was predicted using the STRING database. (b) The interaction between GmSNAT1 and PSYR in plants was verified using BiFC assays. (c) Phenotypic comparison of WT, *GmPSYR1*-OE, and *GmPSYR1*-RNAi plants under cold stress. (d) Representative root scanning images. (e) MDA accumulation in leaves of different lines. (f to j) Quantitative analysis of the root morphological parameters: root volume (f), average root diameter (g), root surface area (h), root length (i), and number of root tips (j). Data are presented as mean ± Sd (*n* = 3 biological replicates). Different letters indicate significant differences (*P* < 0.05, Duncan's test).

Preliminary screening using immunoprecipitation–mass spectrometry (IP–MS) identified potential GmSNAT1-interacting proteins. These were further analyzed using the STRING database (Fig. 6a; Fig. S15). Through this analysis, the interaction between GmSNAT1 and the receptor kinase GmPSYR1 was predicted. *GmPSYR1* is a regulator of root development with putative roles in stress defense (Ogawa-Ohnishi et al. 2022). Consistent with this prediction, RNA-seq data revealed that *GmPSYR1* (*Glyma.10G154300*) transcription levels were upregulated in *GmSNAT1*-OE lines relative to those in M plants (Fig. S16). The interaction between GmSNAT1 and GmPSYR1 was further validated by co-immunoprecipitation (Co-IP), bimolecular fluorescence complementation (BiFC), and yeast 2-hybrid (Y2H) assays (Fig. 6b; Fig. S17).

Given the reported role of *GmPSYR1* in root regulation and defense responses, *GmPSYR1*-OE and RNA interference (RNAi) soybean root lines were generated via hairy root transformation. Phenotypic analysis indicated that robust growth was exhibited by *GmPSYR1*-OE plants under both normal and low-temperature conditions. In contrast, GmPSYR1-RNAi plants performed worse under cold stress, which may be partly attributed to their weaker growth under normal conditions (Fig. 6c). Under cold stress, MDA, O_2_^−^, and H_2_O_2_ levels were significantly lower in *GmPSYR1*-OE plants than in the WT plants. In contrast, these oxidative markers were elevated in *GmPSYR1*-RNAi plants (Fig. 6e; Fig. S18). Antioxidant enzyme activity assays demonstrated that POD activity was significantly higher in *GmPSYR1*-RNAi plants than in WT and *GmPSYR1*-OE plants, under both normal and low-temperature conditions. However, the cold-induced increase in POD activity in *GmPSYR1*-RNAi plants was strongly suppressed by melatonin application (Fig. S18).

Root morphology scanning revealed that root elongation under normal and low-temperature conditions was promoted by *GmPSYR1* overexpression (Fig. 6d and f to j). At low temperatures, the average root diameter of *GmPSYR1*-OE plants was significantly smaller than that of WT and *GmPSYR1*-RNAi plants (Fig. 6g). The average root diameter of *GmPSYR1*-OE plants was significantly increased by exogenous MT; it exceeded that of the WT and *GmPSYR1*-RNAi lines. Although root length in *GmPSYR1*-OE plants was not significantly affected by melatonin, a certain degree of increase in root length was observed in WT and *GmPSYR1*-RNAi plants (Fig. 6i). Under both normal and low-temperature conditions, the number of root tips was significantly higher in *GmPSYR1*-OE plants than in WT plants, whereas a pronounced reduction was observed in *GmPSYR1*-RNAi plants. Under cold stress, the number of root tips in *GmPSYR1*-OE or RNAi plants was barely affected by melatonin; however, a significant increase was observed in the WT plants (Fig. 6j).

Under low-temperature conditions, root length was positively regulated by PSYR. In contrast, the average root diameter was negatively regulated, which may be associated with the suppressive effect of *GmPSYR1* on POD activity. These results demonstrate that an interaction occurs between GmSNAT1 and the receptor kinase GmPSYR1, suggesting that redox homeostasis may be influenced. Root development, particularly root elongation and root-tip formation, may be promoted by *GmSNAT1* through the modulation of *GmPSYR1* expression and endogenous melatonin levels.

## Discussion

Amplified global climate variability has increased the frequency of extreme weather events with detrimental effects on crop production. Cold stress poses a substantial threat to agricultural systems at middle and high latitudes, severely impairing crop growth and development (Clarke and Ronald 2023; Larran et al. 2023). Melatonin is a key endogenous regulator that modulates photosynthesis, osmotic balance, and antioxidant activity in plants (Arnao and Hernández-Ruiz 2019). However, the molecular mechanisms by which melatonin governs cold-stress tolerance in soybeans remain uncharacterized. Building on the foundational work of our research group, an investigation into the mechanistic basis of melatonin-mediated cold tolerance in soybeans was conducted to address this knowledge gap.

Endogenous melatonin biosynthesis in plants involves a cascade catalyzed by tryptophan decarboxylase, tryptamine-5-hydroxylase, SNAT, N-acetylserotonin O-methyltransferase, and COMT (Back et al. 2016). SNAT is the rate-limiting factor in melatonin production (Liu et al. 2022). Suppression of *SNAT* expression in *Arabidopsis* and rice mutants reduces endogenous melatonin levels, thereby severely impairing growth, enhancing abiotic stress sensitivity, and compromising pathogen resistance (Lee et al. 2015; Byeon and Back 2016). Conversely, heterologous overexpression of grape *SNAT* increases melatonin levels and enhances salt tolerance in Arabidopsis (Wu et al. 2021). We established that exogenous melatonin primarily enhanced cold tolerance in soybean by upregulating *GmSNAT1* expression, significantly elevating endogenous melatonin levels.

Enhancing plant resilience to abiotic stresses remains the central goal of modern botany (Zhang et al. 2022). This could be achieved through the strategic coordination of endogenous hormone pathways, including ABA, jasmonic acid, brassinolide, and melatonin (Wang et al. 2019; Waadt et al. 2022). In this study, we established that elevated levels of endogenous melatonin critically protected soybean development under cold stress via dual mechanisms. First, root growth was maintained, consistent with the MT-mediated root promotion observed in *Arabidopsis* (Zuo et al. 2014). This may be related to the fine-tuning of auxin upregulation (Wang et al. 2016). Leaf damage is concurrently mitigated by preserving the chloroplast structure and maintaining photosynthetic efficiency (Zhou et al. 2024). These findings suggest that melatonin is a key regulator that coordinates root–soil interactions and photosynthetic stability, complementing the known ABA-mediated cold adaptation in the common bean (Cheng et al. 2025) and ABA/JA/MT-regulated tolerance in watermelon (Guo et al. 2021). Thus, our understanding of hormonal orchestration during cold adaptation in plants has expanded.

Cold stress induces ROS accumulation in plants, inhibiting their net photosynthetic rate (Apel and Hirt 2004). Here, cold stress led to significant increases in MDA content, REC, and O_2_^−^ levels in soybean seedlings, accompanied by concurrent photosynthetic pigment degradation. Excessive ROS in plants are mitigated by the activation of antioxidant enzyme systems and nonenzymatic antioxidants (Baier et al. 2019). Notably, melatonin enhanced the endogenous antioxidant system, resulting in significantly increased enzyme activity, reduced ROS accumulation, and alleviation of cold-induced damage to the photosynthetic apparatus. Concurrently, melatonin upregulated photosynthesis-related gene expression, increasing the photosynthetic pigment content and net photosynthetic rate. Further insights from transmission electron microscopy revealed that melatonin preserved the chloroplast ultrastructure, reduced the rupture of thylakoid lamellae, accumulated osmiophilic granules, and maintained the integrity of photosynthetic function, reducing the production of ROS. This finding aligns with those of previous reports that indicate that melatonin improves the photosynthetic capacity of tomatoes by increasing the thickness of the chloroplast grana lamella (Debnath et al. 2018).

Ca^2+^ signaling is crucial for cold signal transduction in plants. This process is enhanced by melatonin via Ca^2+^ signaling amplification, which, in turn, activates downstream cold-response mechanisms (Di et al. 2024; Chang et al. 2025). These mechanisms are regulated by CBF-dependent and CBF-independent pathways (Qian et al. 2024). In the present study, the transmission efficiency of the calcium and MAPK signaling pathways under cold stress was improved by melatonin accumulation. This enhancement leads to the activation of cold-regulated gene expression and further modulation of plant hormone pathways, which orchestrate stress defenses via complex signaling networks (Nambara and Van Wees 2021). The expression of genes associated with the auxin, ABA, and ETH pathways was significantly induced by MT or the genetic elevation of endogenous melatonin, coordinating the cold response in soybean. TFs integrate these signals to enhance cold tolerance by regulating the genes involved in membrane stability, antioxidant defense, osmotic regulation, and energy metabolism (Feng et al. 2025). Concurrently, key TFs that govern growth and cold responses have been identified across plant species (Feng et al. 2020; Wang et al. 2023). Transcriptome analyses of exogenous melatonin-treated and endogenous melatonin-overexpressing soybeans revealed that multiple TF families, including WRKY, MYB, NAC, ERF, bHLH, bZIP, and GATA, were induced by melatonin. These TFs participate in the perception and transduction of cold signals and are crucial for melatonin-mediated cold adaptation in soybeans.

Plant small peptides function as novel signaling molecules, with growth, development, and stress responses coordinated via intercellular communication (Zhang et al. 2021). PSY peptides and PSYRs regulate plasma membrane H-ATPase activity to energize active transport, modulating stomatal aperture, cell elongation, and stress adaptation (Falhof et al. 2016; Zhang et al. 2024). PSYR1/2/3 act as redundant negative regulators that suppress growth and activate stress signaling in the absence of PSY; conversely, PSY binding inhibits PSYR signaling and promotes growth (Ogawa-Ohnishi et al. 2022). This switch-like mechanism reflects an evolutionary trade-off between growth and stress tolerance (Nguyen and Kim 2023). In the present study, an interaction between soybean SNAT1, a key enzyme in melatonin biosynthesis, and GmPSYR1 was predicted using IP–MS and transcriptomic analysis. This interaction was experimentally validated using the Y2H, BiFC, and Co-IP assays. The melatonin-mediated activation of *GmPSYR1* transcription was induced by cold stress (Fig. S16). PSYR1 contributed to enhancing the cold tolerance of soybean by improving root development and regulating redox homeostasis. The GmSNAT1–GmPSYR1 interaction may play a role in this process and participate in the regulation of GmPSYR1 cold tolerance by regulating melatonin signaling. Our findings demonstrate a new regulatory axis in which the PSY–PSYR signaling pathway is involved in the regulation of soybean cold tolerance through melatonin-mediated PSYR signal enhancement, which might be influenced by the interaction between GmSNAT1 and GmPSYR1.

## Conclusion

**Figure 7:**
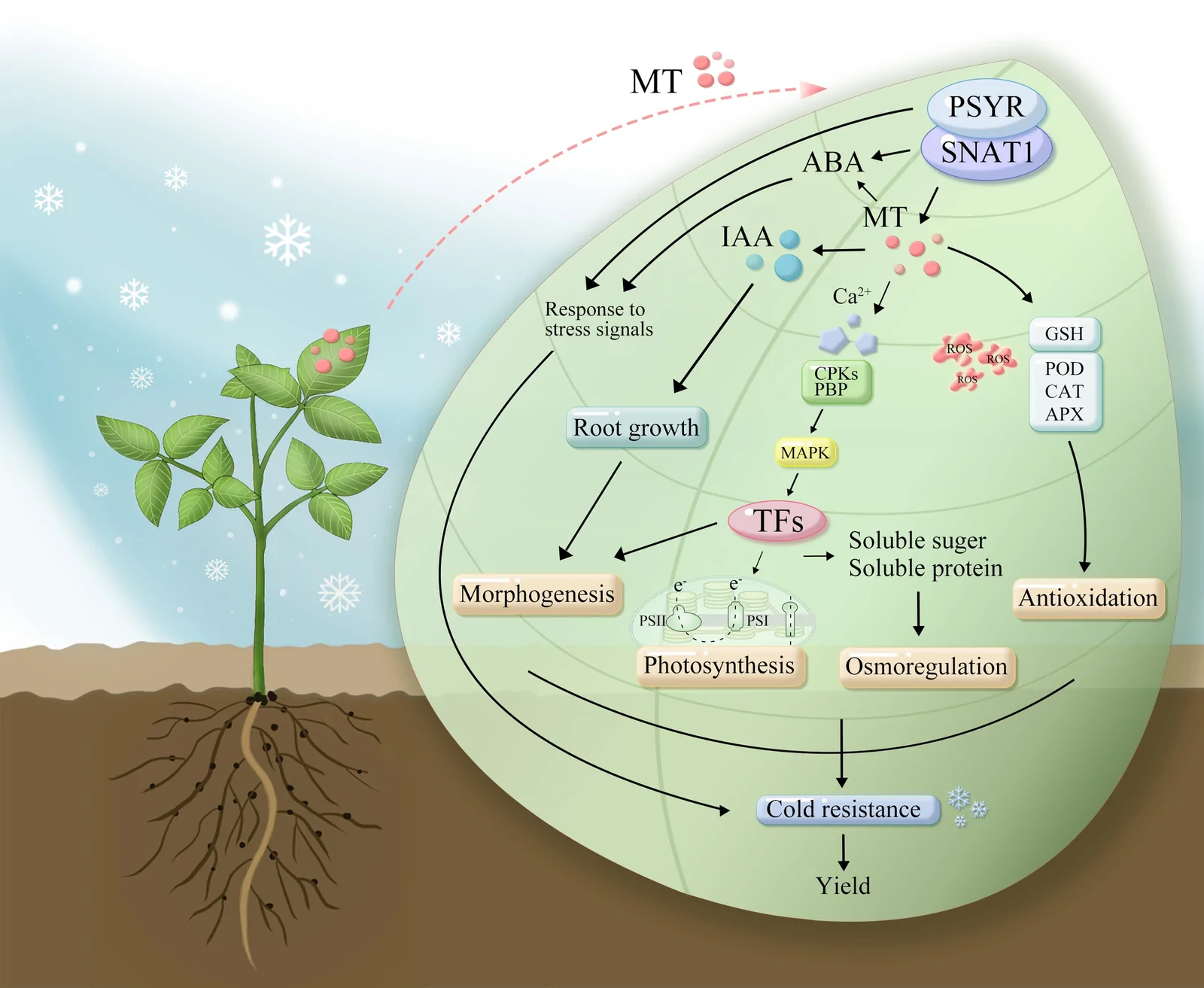
Working model of *GmSNAT1* response to cold stress in soybeans.

We identified *GmSNAT1* as a key positive regulator of cold tolerance in soybean, increasing resilience via a multitiered coordination mechanism (Fig. 7). Under cold stress, endogenous melatonin biosynthesis is stimulated by *GmSNAT1* activation, which amplifies Ca^2+^ signaling and cooperates with the auxin and ABA hormonal networks to orchestrate cold adaptation. The key to cold stress signal transduction involved the PSY–PSYR small peptide signal, and the enhancement of *GmPSYR1* transcription, which is regulated by melatonin, significantly induced root development and redox homeostasis. This might have been influenced by the GmSNAT1–GmPSYR1 interaction, thereby contributing to the enhancement of cold tolerance of soybean. At the transcriptional level, TF networks (eg, WRKY, MYB, NAC, and MYC) are activated by this regulatory system, driving the expression of downstream antioxidant genes to suppress ROS bursts while upregulating photosynthesis-related genes. Consequently, cold-induced inhibition of photosystem II (PSII) activity is mitigated, preserving the function of the electron transport chain and the integrity of the chloroplast ultrastructure. Through this synergy, repair of photosynthetic damage is achieved and holistic protection of photosynthetic function is ensured.

## Materials and methods

### Plant materials

The experimental materials included the cold-sensitive soybean cultivar ‘Nannong 513,’ provided by Nanjing Agricultural University, and the cold-tolerant cultivar ‘Suinong 14,’ provided by the Suihua Branch of the Heilongjiang Academy of Agricultural Sciences. Using ‘Williams 82’ as the genetic background, transgenic plant materials, *GmSNAT1*-OE and GmSNAT1 knockout lines (M), were generated, and hairy root chimeras (PSYR-OE and PSYR-RNAi) were constructed (Supplementary material). Melatonin (powder, molecular weight: 232.28, ≥98% purity; Sigma-Aldrich) was used throughout this study.

### Stress treatments

The experiments were conducted at the National Coarse Cereals Engineering Technology Research Center (Daqing City, Heilongjiang Province, China). Seeds of uniform size and undamaged appearance were surface sterilized with 5% sodium hypochlorite for 1 min, thoroughly rinsed with distilled water, and sown in pots (10 cm diameter × 9 cm height) containing a peat soil/vermiculite (3:1 v/v) substrate. Five biological replicates were included in each treatment. At the V1 growth stage, plants were pretreated with a 100 *μ*mol·L^−1^ melatonin solution applied via foliar spray on dark nights, as determined in preliminary laboratory tests (Ren et al. 2023). Plants were transferred to a climate-controlled growth chamber at 4 °C under a 14-h light/10-h dark photoperiod following melatonin application. After 4 d of cold treatment, phenotype assessments were performed on the plants, and samples were collected for analysis of morphological parameters, photosynthetic characteristics, and chlorophyll fluorescence indices.

Physiological and molecular responses of GmSNAT1-OE and GmSNAT1-M soybean plants ‘WT Williams 82,’ GmSNAT1-OE plants, and GmSNAT1-M plants were evaluated under 2 conditions: normal temperature (25 °C, 4 d) (WT, OE, and M) and 4 °C (4 d) cold stress (WT-C, OE-C, and M-C). Following 4 d of cold treatment, physiological and biochemical indices were measured.

### RNA sequencing

The cold-sensitive soybean cultivar ‘Nannong 513’ was used as the primary test material. Experimental treatments included a normal-temperature control (25 °C), cold-stress treatment (4 °C), and pretreatment with foliar application of 100 *μ*mol·L**^−^**^1^ melatonin in darkness. Six specific treatments were implemented: (i) normal temperature control with water spray (sampled at 0 h, designated h0), (ii) normal temperature with melatonin spray (sampled at 0 h, h0 m), (iii) water spray followed by 3 h at 4 °C (h3), (iv) melatonin spray followed by 3 h at 4 °C (h3 m), (v) water spray followed by 24 h at 4 °C (h24), and (vi) melatonin spray followed by 24 h at 4 °C (h24 m). Three biological replicates were included for each treatment, with leaf sampling for transcriptome analysis conducted at 0, 3, and 24 h postinitiation of cold exposure. Concurrently, **‘**WT Williams 82,’ GmSNAT1-OE plants, and GmSNAT1-M plants were evaluated under 2 conditions: normal temperature (WT, OE, and M) and 4 °C (4 d) cold stress (WT-C, OE-C, and M-C). The 6 treatments were replicated thrice for transcriptome sequencing. Transcriptome analyses, including RNA sequencing, differential gene expression profiling, enrichment pathway analysis, and key differential gene screening, were performed by Hangzhou Lianchuan Biotechnology Co., Ltd.

The first set of transcriptome data was previously validated (Ren et al. 2023). To verify the reliability of the transcriptome data, 9 DEGs were randomly selected from the second transcriptome dataset for qRT-PCR analysis. Although differences in gene expression were observed (Fig. S19), the RT-qPCR results for the 9 DEGs were consistent with the transcriptomic trends. This indicated that the transcriptomic data of this study were reliable and suitable for further analysis.

### Determination of plant morphological indices

Paraffin sectioning was performed following a previously described method with modifications (Fan et al. 2018). The first 3 compound leaves of each treatment were collected, and leaves (0.5 cm) were cut from the same part to avoid veins. Samples were subjected to vacuum infiltration in an FAA fixative (formalin/acetic acid/ethanol, 1:1:18), followed by fixation at 4 °C for 24 h to preserve tissue integrity. The fixed materials were then dehydrated using a graded ethanol series (30%, 50%, 70%, 85%, 95%, and 100%), cleared in xylene to eliminate residual ethanol, and embedded in paraffin. Serial longitudinal sections (10 *μ*m in thickness) were prepared using a Leica rotary microtome. Following dewaxing and rehydration, the sections were stained with 0.1% toluidine blue and visualized using bright-field optics (Nikon Eclipse E100).

Analysis of the mesophyll cell ultrastructure was performed following a previously described method with modifications (Yin et al. 2015). Trifoliate leaves from each treatment were rinsed thrice in distilled water and blotted dry. Transverse leaf segments (0.2 cm width) containing midveins were excised from standardized positions, with 3 biological replicates per treatment, and immediately transferred within 60 s to a primary fixative (2.5% glutaraldehyde in 0.1 m phosphate buffer, pH 7.2) under ventilated conditions. The samples were vacuum infiltrated, covered with lint-free mesh to ensure submersion, and sealed in light-proof containers with parafilm and aluminum foil for 24 h at 4 °C. Dehydration was performed using a graded ethanol series (30%, 50%, 70%, 80%, 95%, and 2× 100%) at 25 °C for 1 h per step. Embedding was performed using SPI-Pon 812 epoxy resin via progressive acetone-resin transitions: 3:1 (37 °C, 3 h), 1:1 (37 °C, overnight), and 1:3 (37 °C, 4 h), followed by pure resin infiltration (37 °C, 6 h). Specimens were oriented in silicone molds, polymerized at 60 °C for 48 h, and sectioned to a thickness of 60 to 80 nm using a diamond knife (Leica UC7). Sections collected on 150-mesh copper grids were poststained with 2% uranyl acetate and Reynolds lead citrate before transmission electron microscopy imaging (Hitachi HT7800/HT7700, 80 kV).

### Determination of plant photosynthetic index

The main photosynthetic indices, including the net photosynthetic rate, stomatal conductance, transpiration rate, and intercellular carbon dioxide (CO_2_) concentration, were measured using the Li6400 photosynthesis system. Measurements were performed on the first 3 leaves of the soybean seedlings, with 3 replicates for each treatment.

The chlorophyll content was determined using a handheld SPAD-502 chlorophyll meter. Measurements were performed at the same position on the first compound leaf of the soybean plants, and the average SPAD value was calculated from 3 measurement points per leaf.

The actual light energy conversion efficiency of PSII was measured using the PlantView 230F modulated chlorophyll fluorescence in an in vivo imaging system. After 30 min of dark treatment, the maximum light-energy conversion efficiency of PSII was determined. Fluorescence images were collected in triplicates for each treatment.

### Determination of plant physiological indices

Five leaf segments of 1.5 cm in length and 0.5 cm in width were cut and placed in a 15 mL centrifuge tube, to which 10 mL of distilled water was added. The initial conductivities (E0) were measured using a conductivity meter (DDS-11A). After shaking at 180 rpm for 4 h at 25 °C, conductivity (E1) was recorded. The mixture was boiled for 30 min and cooled to 25 °C, and its conductivity (E2) was measured. Relative conductivity was calculated as the ratio of (E1–E0) to (E2–E0), with 5 replicates for each treatment.

Prior to sampling, plant tissues were rinsed with ultrapure water to prevent exogenous contamination. Samples were supplemented with 25 mL of acetonitrile solution at 4 °C. The mixture was homogenized in an ice bath at 10,000 rpm for 2 min, followed by the addition of 4 g anhydrous magnesium sulfate, 1 g sodium chloride, 1 g sodium citrate, and 0.5 g disodium citrate sesquihydrate. The mixture was vortexed for 1 min and centrifuged at 10,000 rpm (4 °C) for 5 min. A 5 mL aliquot of the supernatant was transferred to a 15 mL centrifuge tube containing 150 mg PSA, 150 mg C18EC, and 900 mg anhydrous magnesium sulfate and vortexed for 1 min. After centrifugation at 10,000 rpm for 5 min at 4 °C, 2 mL of the supernatant was dried under nitrogen, reconstituted to 0.5 mL with formic acid:water (5:95), and analyzed using liquid chromatography–tandem mass spectrometry (Cao et al. 2022). ABA content was analyzed in negative ion mode, whereas indole acetic acid and melatonin were analyzed in positive ion mode using a chromatographic column (Atlantis T3, 2.1 × 150 mm, 3 *μ*m).

The biochemical assays were performed using commercial kits (Grace Biotechnology, Suzhou, China). MDA content in leaves was determined via the thiobarbituric acid reaction. Concurrently, the concentration of Ca^2+^ was measured using colorimetric detection. Hydrogen sulfide and reduced glutathione levels were assessed using specific fluorometric methods. The activities of the antioxidant enzymes, including SOD (EC 1.15.1.1), POD (EC 1.11.1.7), and CAT (EC 1.11.1.6), were quantified using UV–Vis spectrophotometry. Simultaneously, H_2_O_2_ content was determined using titanium sulfate precipitation. All assays were performed according to the manufacturer's protocol, with 3 technical replicates per biological sample.

### Reactive oxygen staining of plant tissues

H_2_O_2_ accumulation was visualized using 3,3′-diaminobenzidine (DAB) staining, following the method previously described with modifications (Ivanchenko et al. 2013). Leaf samples collected from standardized positions were vacuum infiltrated with a 0.5% DAB solution (pH 3.8) and incubated in the dark at 25 °C for 12 h. Chlorophyll was removed using a decolorization solution (ethanol:acetic acid, 7:3 v/v) at 80 °C until the tissues were bleached. Subsequently, the samples were mounted in 50% glycerol for bright-field imaging. For O_2_^−^ detection, nitro blue tetrazolium (NBT) staining was used. Leaves were immersed in 1% NBT solution prepared in 0.1 m phosphate buffer (pH 7.8) and subjected to the same incubation and decolorization conditions as those used for DAB staining for 24 h.

For in situ imaging of ROS in roots, 20 mm apical segments were longitudinally sectioned. For H_2_DCFDA staining, a 10 mm stock solution in DMSO was diluted to 50 *μ*m with 10 mm Tris-HCl buffer (pH 7.4). The root segments were then incubated in this working solution at 25 °C in the dark for 60 min. For dihydroethidium (DHE) staining, the root tissues were incubated for 15 min in a 15 *μ*m working solution, which was prepared by diluting a 10 mm stock in DMSO with the same Tris-HCl buffer (pH 7.4). After incubation, both fluorescent probes were rinsed thrice with the buffer. Confocal imaging was conducted using a Leica TCS SP8 microscope, with excitation at 488 nm and emission at 500 to 550 nm for H_2_DCFDA and excitation at 535 nm and emission at 560 to 620 nm for DHE. Z-stack images were acquired using a 20× objective with 2 *μ*m steps.

### Verification of the interaction between GmSNAT1 and PSYR

The experiments included western blotting, IP-MS, Co-IP, BiFC verification, and Y2H assays. Given the complexity of the experimental procedures, the specific operational details are included in the Supplementary material.

### Data processing and analysis

All the data were collated in Microsoft Excel 2019, and statistical analyses were performed using SPSS 20.0. One-way analysis of variance was used to assess differences, followed by multiple comparisons using the least significant difference test. Statistical significance was set at *P* < 0.05. Graphs were generated using Origin 2021, and figures were further processed using Adobe Illustrator 2021.

## Supplementary Material

kiag516_Supplementary_Data

## Data Availability

The data supporting the findings of this study are available within the article and its supplementary materials.
