# Supplementary material for: GmSNAT1 enhances cold tolerance in soybean by promoting melatonin biosynthesis and interacting with GmPSYR1
Source: Plant Physiol. 2026 Jul 15;202(1):kiag516. doi: 10.1093/plphys/kiag516 (PMC13591401; doi:10.1093/plphys/kiag516)
Supplement: Supplementary file 1 [file kiag516_supplementary_data.doc]

Fig.S1 Anatomical structure of seedling leaves under room temperature control. CK: control. MT: melatonin treatment.

Fig.S2 Apparent data of seedling roots under low temperature stress. CK: control. MT: melatonin treatment. C: 4 °C cold treatment. C-MT: melatonin pretreatment + 4 °C cold treatment. Data are presented as mean ± SD (n = 3 biological replicates). Significant differences are indicated by different letters (p < 0.05, Duncan's test).

Fig.S3 Gene ontology (GO) enrichment of differentially expressed genes (DEGs) in melatonin-treated and control seedlings under cold stress. (C: Control; MT: Melatonin treatment; h3/h24: 3/24 h cold exposure).

Fig.S4 Kyoto Encyclopedia of Genes and Genomes enrichment of DEGs (C-MT vs. C).

Fig.S5 Ca^2+^ content in seedling leaves under low temperature stress.CK: control. MT: melatonin treatment. C: 4 °C cold treatment. C-MT: melatonin pretreatment + 4 °C cold treatment. Data are presented as mean ± SD (n = 3 biological replicates). Significant differences are indicated by different letters (p < 0.05, Duncan's test).

Fig.S6 Heatmap of photosynthesis-related DEG expression.

Fig.S7 Heatmap of cold-response and antioxidant DEG expression.

Fig.S8 Antioxidant activity of seedlings under low temperature stress. GSH: glutathione. GSSG: oxidized glutathione. CK: control. MT: melatonin treatment. C: 4 °C cold treatment. C-MT: melatonin pretreatment + 4 °C cold treatment. Data are presented as mean ± SD (n = 3 biological replicates). Significant differences are indicated by different letters (p < 0.05, Duncan's test).

Fig.S9 *GmSNAT1* regulates root growth of soybean seedlings. WT: wild type; M: *GmSNAT1* knockout plants; OE: *GmSNAT1* overexpression plants. Data are presented as mean ± SD (n = 3 biological replicates). Significant differences are indicated by different letters (p < 0.05, Duncan's test).

Fig.S10 Enrichment analysis of differentially expressed genes (DEGs) involved in cold-responsive and antioxidant pathways.

Fig.S11 Antioxidant activity of different soybean materials under low temperature stress. WT: Wild type. OE: *SNAT1* overexpression material. M: *SNAT1* knockout material. Data are presented as mean ± SD (n = 3 biological replicates). Significant differences are indicated by different letters (p < 0.05, Duncan's test).

Fig.S12 *GmSNAT1* knockout plant reverse complementation test. WT: Wild type. M: *SNAT1* knockout material. M-MT: *SNAT1* knockout material-melatonin treatment(Melatonin concentration：100 μmol/L). Data are presented as mean ± SD (n = 3 biological replicates). Significant differences are indicated by different letters (p < 0.05, Duncan's test).

Fig.S13 Antioxidant activity of plants in melatonin complementation test (*SNAT1* knockout material). WT: Wild type. M: *SNAT1* knockout material. M-MT: *SNAT1* knockout material-melatonin treatment. Data are presented as mean ± SD (n = 3 biological replicates). Significant differences are indicated by different letters (p < 0.05, Duncan's test).

Fig.S14 Gas exchange parameters of plants in melatonin complementation test (*SNAT1* knockout material). WT: Wild type. M: *SNAT1* knockout material. M-MT: *SNAT1* knockout material-melatonin treatment. Data are presented as mean ± SD (n = 3 biological replicates). Significant differences are indicated by different letters (p < 0.05, Duncan's test).

Fig.S15 Potential GmSNAT1-interacting proteins were identified using immunoprecipitation-mass spectrometry (IP-MS).

Fig.S16 Enrichment analysis of differentially expressed genes (DEGs) associated with PSYR-mediated small peptide signaling is presented. A) Plant small peptide related differential gene enrichment heat map. B) Expression *GmPSYR1* (*Glyma.10G154300*) in different materials under cold stress (WT: wild-type, OE: GmSNAT1-OE lines, M: GmSNAT1 knockout lines.) C) GmPSYR1 was induced by melatonin under cold stress. CK: control. MT: melatonin treatment. C : 4 ° C cold treatment. C-MT: melatonin pretreatment + 4 ° C cold treatment.

Fig.S17 GmSNAT1-PSYR interaction. A) Yeast two-hybrid (Y2H) assay confirming the GmSNAT1-PSYR interaction is displayed. B) Co-immunoprecipitation (Co-IP) validation of the GmSNAT1-PSYR interaction is provided.

Fig.S18 Different *GmPSYR1* materials O_2_^·-^and H_2_O_2_ content and antioxidant enzyme activity. WT: wild type; OE: *GmPSYR1*-OE plants; RNAi: *GmPSYR1*-RNAi plants. Data are presented as mean ± SD (n = 3 biological replicates). Significant differences are indicated by different letters (p < 0.05, Duncan's test).

Fig. S19 qRT-PCR to verify the accuracy of transcriptome data.

**Experimental method supplement**

**Construction of GmSNAT1 overexpression transgenic material**

Through homologous alignment analysis with Arabidopsis thaliana and other plants, eight N-acetyltransferase-encoding genes were identified in soybean. The *GmSNAT1* (*Glyma.09g272400*) gene with relatively high expression level in soybean plants was selected as the target gene for subsequent research. PCR amplification was performed using specific primers. The results of PCR product electrophoresis showed that the size of the amplified fragment was consistent with the size of the target gene fragment. The PCR product was recovered and sequenced correctly. The *GmSNAT1* overexpression vector was constructed by homologous recombination. The PCR identification of bacterial solution was carried out by using vector primers. The results of electrophoresis showed that the PCR products of 8 bacterial solutions all appeared at about 551 bp, which was consistent with the size of the target fragment. In summary, the BS-SNAT1-3xflag-2-35s-gfp-tnos expression vector was constructed correctly.

Bacterial liquid electrophoretic detection

The constructed BS-SNAT-3xflag-2-35s-gfp-tnos vector was transformed into Williams 82 background material by Agrobacterium-mediated method. The positive transgenic plants were preliminarily screened by Bar test strip and target fragment PCR technology. In order to screen homozygous transgenic plants, Western Blot was performed on T3 transgenic lines. The results showed that SNAT1-FLAG protein was successfully identified in both OE-1 and OE-2 lines. Moreover, the expression level of *GmSNAT1* and the content of melatonin in leaves of OE-1 and OE-2 lines were significantly higher than those of wild type. In summary, the results showed that OE-1 and OE-2 were genetically stable *GmSNAT1* overexpression transgenic lines.

Agrobacterium-mediated transformation of soybean cotyledon nodes

**Construction of GmSNAT1 gene knockout material**

In order to further clarify the function of *GmSNAT1* in regulating soybean cold tolerance and reveal the mechanism of endogenous melatonin regulating plant cold tolerance, this study used CRISPR / Cas9 system to construct *GmSNAT1* gene editing materials. The construction of GmSNAT1 gene knockout expression vector A total of two target sites were designed, and the target sequence information is shown in the following table. Since the coding sequences of *Glyma.09G272400* and *Glyma.18g216900* are highly similar, in order to prevent functional redundancy, the target is designed on the conserved sequences of the two sequences. Target 1 is located on the fourth exon of the two genes, and target 2 is located on the sixth exon of the two genes. The tRNA + target + gRNA skeleton was used as a unit, and the two targets were connected in series to construct a vector (ZMPL). The CaMV35S promoter encodes the Cas9 protein, and atu26 initiates sgRNA expression.

Table. The target site of *GmSNAT1* gene by Grispr/Cas9

| Name of target point | target sequence |
| --- | --- |
| GmSNAT1-T1 | GTGGAAAAGACAGAACCCGA TGG |
| GmSNAT1-T2 | GGCATGGTCAGACGTAGCAC GGG |

After sequencing, the successfully constructed plasmid was transformed into Agrobacterium EHA105 for stable genetic transformation. Willianms82 was used as the receptor, and the editing materials were obtained by Agrobacterium transformation. PCR was used to detect and the editing materials (T3) were further sequenced . Compared with the wild type, the target sequence of M-1 showed base deletion, and the target sequence of M-2 showed base mutation. At the same time, the expression level of *GmSNAT1* gene and melatonin content in leaves of M-1 and M-2 lines were determined to obtain *GmSNAT1* knockout plants (see Fig.3 for verification).

**GmPSYR1-OE and GmPSYR1-RNAi Vector construction**

The soybean (*Williams 82*) was used as experimental material. The SGN-VIGS tool (https://vigs.solgeno mics.net/) (accessed on October 15, 2025) was used to identify the silent fragments of *GmPSYR1*(325bp). SnapGene was adopted to design primers, and the RNAi vector (pNC-Cam3304- RNAi), and OE vector (pNC-Cam1304-MCS35S), derived from NC Biotech (ncloning.com), were utilized to construct target vectors (Yan et al., 2023). The Nimble cloning kit (NC001) of NC Biotech was employed to construct *GmPSYR1*-OE, *GmPSYR1*-RNAi vectors using the objective fragment of the target gene with a universal adapter (Yan et al., 2020). The vectors were then transformed into E. coli (DH5 α) competent cells by heat shock method. After kanamycin resistance screening, the positive strains were obtained and propagated, and the recombinant plasmids were extracted. Then RNAi empty vector, OE empty vector, *GmPSYR1*-OE, *GmPSYR1*-RNAi recombinant vectors were transformed into Agrobacterium rhizogenes strain K599 by heat shock method.

*GmPSYR1*-OE vector and *GmPSYR1*-RNAi vector agrobacterium colony PCR gel electrophoresis (OE: 1035bp; RNAi: 325bp)

DNA sequencing of GmPSYR1-OE vector GmPSYR1

**Hairy root chimera culture**

Hairy root chimera culture was conducted according to the method of Xia with some modifications(Xia et al., 2024). The surface-sterilized soybean (Williams 82) seeds were germinated in vermiculite. After the cotyle dons were unearthed, the wound was formed by cutting the hypocotyl of the seedling at a 45^◦^ angle, which was soaked in the infection solution with Agrobacterium. The sterilized filter paper was spread on a disposable sterile culture dish, and soaked with 10 mL sterile water. The seedling wounds were placed upward in a Petri dish and then co-cultured for 24h under dark conditions. Soybean plants were transferred to black pots containing vermiculite (3–6 mm) to induce hairy roots. The vermiculite was kept moist. When the cotyledons were expanded, 1/2 Hoagland nutrient solution was added, and adventitious roots were cleared regularly. After 15 days, the hairy roots were induced to grow in large numbers, and the chimeric plants formed ternate compound leaves. Root samples were subjected to qRT-PCR analysis to identify soybean plants with targeted gene silence or over-expression for further study.

**Total Protein Extraction and Western Blotting**

The experimental procedure was performed based on the method described by Flury with appropriate modifications (Flury et al., 2013). An appropriate amount of leaves from the OE material was ground into a powder in liquid nitrogen. Subsequently, 600 μL of IP Lysis Buffer containing Protease Inhibitor Cocktail (1×) was added, and the mixture was vortexed to achieve a homogenized state. The homogenate was allowed to stand in an ice bath for 30 min, with vortexing for 15 s every 10 min. After that, centrifugation was carried out at 4 °C and 15,000 r/min for 10 min, and the supernatants was collected. The centrifugation step was repeated, and the resulting supernatants was used as the total protein, which was then stored at -80 °C.

Following the addition of samples, electrophoresis was initiated. The concentrated gel was run at 80 V for 30 min, and the separation gel was run at 120 V for 60 min. After electrophoresis, the gel was taken out and placed in pure water for subsequent use. A PVDF membrane with the same size as the separation gel was cut, marked, activated in methanol for 30 s, and then equilibrated in equilibration buffer. On the negative electrode (-) side of the membrane clip, sponge, gel, PVDF membrane, and sponge were placed in sequence, fixed, and then the assembly was put back into the rapid wet transfer instrument, with an appropriate program selected for the transfer process. After the transfer was completed, the membrane was taken out, washed in PBS for 5 min, and then transferred to an automatic Western hybridization instrument. Appropriate primary antibody, secondary antibody, and washing solution were added to the corresponding sample tanks of the hybridization instrument, and exposure was performed upon completion of the operation. Equal volumes of ECL luminescent liquid A and liquid B were mixed thoroughly and stored in the dark for later use. The membrane was taken out from PBS, and excess water stains were removed using filter paper. The membrane was placed on the exposure stage with the protein side facing up, and the mixed luminescent liquid was applied to cover the entire membrane. First, a white light image was captured, followed by a fluorescence image; these two images were then merged and saved.

**CoIP assay**

Protein A/G beads were retrieved, and 40 μL aliquots were dispensed into two 1.5 mL EP tubes. Pre-cooled PBS (0.5 mL) was added to resuspend the beads, followed by centrifugation at 500 rpm and 4 °C for 1 min. The beads were separated from the solution, and the supernatants was discarded. This washing procedure was repeated twice, resulting in a total of three washes, with 30 μL PBS finally added to resuspend the beads.

Target antibody (10 μg) was added, and the mixture was incubated at room temperature for 1 h with gentle mixing. After centrifugation at 500 rpm and 4 °C for 1 min, the beads were separated from the solution, and the supernatants was discarded. Pre-cooled PBS (0.5 mL) was added to resuspend the beads, which were then centrifuged at 500 rpm and 4 °C for 1 min. The beads were separated, the supernatants was discarded, and this sequence of steps was repeated to achieve a total of three washes.

The total protein solution was added, and the mixture was incubated overnight (approximately 16 h) at 4 °C with gentle mixing. Following centrifugation at 500 rpm and 4 °C for 1 min, the beads were separated from the solution, and the supernatants was discarded. Pre-cooled PBS (0.5 mL) was added, and centrifugation was performed at 500 rpm and 4 °C for 1 min. The beads were separated from the solution, the supernatants was discarded, and this washing step was repeated five times.

IP Lysis Buffer (45 μL) and SDS Sample Buffer (4×, 15 μL) were added, and the mixture was heated in a metal bath at 100 °C for 5 min. After centrifugation at 15,000 rpm for 5 min, the supernatants was collected and designated as the IP group. A 45 μL aliquot of the total protein solution was taken, mixed with 15 μL SDS Sample Buffer (4×), and heated in a metal bath at 100 °C for 5 min. Subsequent detection was performed using Western Blot (Liu et al., 2017).

**Mass spectrometry detection**

Protein bands of interest from the SDS-PAGE gel were excised into small pieces approximately 1 mm³ in size. Double-distilled water was added to immerse the gel pieces, which were then shaken for 10 min before the washing solution was aspirated. The gel pieces were subsequently immersed in a 50% ACN/100 mM NH₄HCO₃ (pH 8.0) solution, shaken for 10 min, and the washing solution was removed. This washing process was repeated three times.

Pure ACN (100%) was added to immerse the gel pieces, which were shaken for 10 min. After the washing solution was aspirated, the gel pieces were dried in a vacuum dryer. A 10 mM DTT/50 mM NH₄HCO₃ (pH 8.0) solution was added to the dried gel pieces, which were then incubated at 56 °C for 1 h to facilitate the reduction reaction. Following this, the supernatants was aspirated. Subsequently, a 55 mM iodoacetamide/50 mM NH₄HCO₃ (pH 8.0) solution was added, and the reaction was allowed to proceed at room temperature in the dark for 30 min.

Pure ACN (100%) was added, and the mixture was shaken for 20 min. The supernatant was aspirated, and the gel pieces were dried. An appropriate amount of trypsin was added to the gel pieces, followed by sufficient 50 mM NH₄HCO₃ solution to completely cover the gel. The samples were incubated overnight at 37 °C for enzymatic digestion.

A 60% ACN/5% formic acid solution was then added to extract the peptides. The mixture was ultrasonically shaken for 10 min, centrifuged, and the supernatants was transferred to a new centrifuge tube. After this extraction process repeated twice, the supernatants were combined and dried in a centrifugal concentrator. The resulting peptides were desalted using a C18 column and stored frozen at -20 °C until detection.

Mass spectrometry detection was performed using an Orbitrap Astral mass spectrometer coupled with a Vanquish NEO liquid chromatography-mass spectrometry system. Peptide samples were dissolved in loading buffer, then aspirated by an autosampler and loaded onto an analytical column (150 μm × 150 mm, C18, 2 μm, 100Å) for separation. Mass spectrometric data were acquired in either DIA or DDA mode.

**BiFC assays**

Firstly, Nicotiana benthamiana plants were cultured for one month to be used in the experiment. The constructed vector plasmids were transferred into Agrobacterium tumefaciens (GV3101) via electroporation and incubated at 30 °C for 2 days. Agrobacterial colonies were scraped from the solid culture plates using an inoculating loop and then cultured in YEB liquid medium containing the corresponding antibiotics at 170 rpm for 1 h. The bacterial culture was centrifuged at 4,000 rpm for 4 min, and the supernatants was discarded. The bacterial cells were resuspended in a 10 mM MgCl₂ solution containing 120 μM acetosyringone (AS), and the OD₆₀₀ value was adjusted to approximately 0.6.

Healthy N. benthamiana plants with good growth status were selected, and the bacterial suspension was injected into the lower epidermis of N. benthamiana leaves using a 1 mL syringe without a needle, followed by labeling. The injected N. benthamiana plants were cultured under low light conditions for 2 days. The labeled leaves injected with Agrobacterium were harvested and prepared into slides. These slides were observed under a laser confocal microscope, and images were captured (Ren et al., 2021).

**Y2H assays**

A single colony of AH109 was picked from the YPDA solid plate and inoculated into 50 mL of 2×YPDA liquid medium, followed by incubation at 30 °C with shaking at 225 rpm for 12 h. Cells were harvested by centrifugation at 700 × g for 5 min at room temperature, and the supernatants was discarded. The bacterial cells were resuspended in sterile water, mixed thoroughly, and centrifuged again at 700 × g for 5 min at room temperature to collect the cells, with the supernatants discarded. A PEG/LiAc master mix solution was prepared by resuspending the cells in 2.5 mL of sterile water.

In a sterile 1.5 mL centrifuge tube, 1 μg of each combination plasmid was added. Subsequently, 300 μL of the PEG/LiAc master mix and 100 μL of the resuspended yeast cells were added, and the mixture was gently pipetted to ensure thorough mixing. Heat shock was performed at 42 °C for 45 min, with mixing every 15 min. After heat shock, cells were collected by centrifugation at 700 × g for 5 min at room temperature, and the supernatants was discarded. Each transformation mixture was resuspended in 300 μL of sterile water, gently pipetted to mix, and then plated onto the corresponding defective screening plates. These plates were incubated at 30 °C for 3- 5 days.

Colonies from the aforementioned plates were picked and added to 100 μL of sterile water, followed by dispersion and thorough mixing. Each bacterial suspension was spotted onto DDO, TDO/X, and QDO/X solid media, with 5 μL of the bacterial suspension applied per spot. The plates were incubated upside down at 30 °C for 3- 5 days until colonies grew.

**Reference**

**Flury P, Klauser D, Boller T, Bartels S** (2013) MAPK Phosphorylation Assay with Leaf Disks of Arabidopsis. Bio-protocol **3:** e929

**Liu W, Sun Q, Wang K, Du Q, Li WX** (2017) Nitrogen Limitation Adaptation (NLA) is involved in source-to-sink remobilization of nitrate by mediating the degradation of NRT1.7 in Arabidopsis. New Phytologist **214:** 734-744

**Ren YR, Zhao Q, Yang YY, Zhang R, Wang XF, Zhang TE, You CX, Huo HQ, Hao YJ** (2021) Interaction of BTB-TAZ protein MdBT2 and DELLA protein MdRGL3a regulates nitrate-mediated plant growth. Plant Physiology **186:** 750-766

**Xia T, Zhu X, Zhan Y, Liu B, Zhou X, Zhang Q, Xu W** (2024) The white lupin trehalase gene LaTRE1 regulates cluster root formation and function under phosphorus deficiency. Plant Physiology

**Yan P, Tuo D, Shen W, Deng H, Zhou P, Gao X** (2023) A Nimble Cloning-compatible vector system for high-throughput gene functional analysis in plants. Plant Communications **4:** 100471

**Yan P, Zeng Y, Shen W, Tuo D, Li X, Zhou P** (2020) Nimble Cloning: A Simple, Versatile, and Efficient System for Standardized Molecular Cloning. Frontiers in Bioengineering and Biotechnology **7**
